# HF Radar Sea-echo from Shallow Water

**DOI:** 10.3390/s8084611

**Published:** 2008-08-06

**Authors:** Belinda Lipa, Bruce Nyden, Don Barrick, Josh Kohut

**Affiliations:** 1 Codar Ocean Sensors, 125 La Sandra Way, Portola Valley, CA 94028 USA; 2 Codar Ocean Sensors, 1914 Plymouth Street, Mountain View, CA 94043 USA; E-mail: bruce@codar.com; don@codar.com; 3 Coastal Ocean Observation Lab., Rutgers University, New Brunswick, NJ 08901 USA; E-mail: kohut@marine.rutgers.edu

**Keywords:** HF radar oceanography, wave measurement, remote sensing

## Abstract

HF radar systems are widely and routinely used for the measurement of ocean surface currents and waves. Analysis methods presently in use are based on the assumption of infinite water depth, and may therefore be inadequate close to shore where the radar echo is strongest. In this paper, we treat the situation when the radar echo is returned from ocean waves that interact with the ocean floor. Simulations are described which demonstrate the effect of shallow water on radar sea-echo. These are used to investigate limits on the existing theory and to define water depths at which shallow-water effects become significant. The second-order spectral energy increases relative to the first-order as the water depth decreases, resulting in spectral saturation when the waveheight exceeds a limit defined by the radar transmit frequency. This effect is particularly marked for lower radar transmit frequencies. The saturation limit on waveheight is less for shallow water. Shallow water affects second-order spectra (which gives wave information) far more than first-order (which gives information on current velocities), the latter being significantly affected only for the lowest radar transmit frequencies for extremely shallow water. We describe analysis of radar echo from shallow water measured by a Rutgers University HF radar system to give ocean wave spectral estimates. Radar-derived wave height, period and direction are compared with simultaneous shallow-water in-situ measurements.

## 1. Introduction

HF radar systems are widely used internationally to provide continuous monitoring of ocean waves and currents for a large range of environmental conditions.

Within the US, coastal ocean current mapping with HF radar has matured to the point where it is now considered an important component of regional ocean observing systems. A mid-Atlantic HF radar network now provides high resolution coverage within five localized networks, which are linked together to cover the full range of the mid-Atlantic coastal ecosystem. Similar regional networks around the US coastline are being formed into a national HF radar network.

While much of the focus of these networks until now has been on offshore current mapping observations, a longer-term objective is to develop and evaluate near-shore measures of waves and currents. These investigations aim to understand the interaction of waves in the shallow coastal waters and how energy is transformed into the creation of dangerous rip currents along the New-Jersey/Long-Island shorelines. Rutgers University radars cover these coastal regions at multiple frequencies from 4.5 to 25 MHz. Their echoes contain information on both currents and waves from deep water up into the shallow coastal zone, providing an excellent archive for such studies. This paper describes the analysis of both simulated and measured radar echo to demonstrate the effect of shallow water on radar observations and their interpretation.

Radar sea-echo spectra consist of dominant first-order peaks surrounded with lower-energy second-order structure. Analysis methods presently in use assume that the waves do not interact with the ocean floor, see [1, 2, 3] for phased-array-antenna beam-forming systems; and [4] for systems with compact crossed-loop direction-finding antennas, such as the SeaSonde.

The assumption of deep water is often invalid close to the coast and for broad continental shelves, and is particularly inadequate to describe the second-order sea-echo used to give information on ocean waves., as second-order echo is often visible above the noise only for close ranges. To interpret this echo correctly, we show that the effects of shallow water must be taken into consideration.

In Section 2, we give the basic equations describing radar echo from shallow water, expanding on the previous description given in [5]. In Section 3, simulations are used to illustrate the effects of shallow water on waveheight, Doppler shifts and spectral amplitudes in radar sea-echo spectra, to investigate limits on the existing theory and to define depth limits at which shallow-water effects must be included in the analysis. The effects of shallow water on the radar spectrum are illustrated using measured spectra. In Section 4, methods are applied to the interpretation of measured radar echo from a Rutgers University radar to produce wave directional spectral estimates, which are compared with wave observations from a bottom-mounted Acoustic Doppler Current Profiler (ADCP) moored in the second radar range cell.

## 2. Radar spectral theory

It follows from the solution of the equations of motion and continuity that long ocean waves are more affected by shallow water. We define the depth at which waves interact with the ocean floor by the approximate relation:

(1) d/L≤1/8

where *d* is the water depth and *L* is the dominant ocean wavelength. The deep-water analysis must be modified to allow for shallow-water effects in the coupling coefficients, the dispersion equation refractive effects on wave direction, and the directional ocean wave spectrum itself. We only consider water of sufficient depth that effects of wave energy dissipation such as breaking and bottom friction may be ignored; thus we operate in the linear wave transformation regime. As a general rule, this assumption is valid when the water depth is greater than 5% of the deep-water wavelength.

Applying the lowest-order shallow-water dispersion equation to first-order backscatter from the sea gives the following equations for ${\tilde{k}}_{s}^{1}$ , the first-order spatial wave vector and $\omega_{s}^{1}$ , the temporal wavenumber of the ocean waves in shallow water producing the backscatter. In this document, a subscript or superscript *s* indicates a shallow-water variable; its absence indicates a deep-water variable.

(2) $$\begin{array}{lll} {\tilde{k}}_{s}^{1} & = & -2{\tilde{k}}_{0}\\ \omega_{s}^{1} & = & m'\omega_{B} \end{array}$$

where *k̃*_0_ is the radar wave vector, of magnitude *k*_0_ , *ω_B_* and is the Bragg resonant frequency in shallow water which is given by:

(3) $$\omega_{B}=\sqrt{2gk_{0}\tan\text{h}(2k_{0}d)}$$

with *g* the gravitational constant. The analogous relations for second-order backscatter are:

(4) $$\begin{array}{l} {\tilde{k}}_{s}+{\tilde{k}}_{s}^{'}=-2{\tilde{k}}_{0}\\ \omega_{s}^{2}=m\sqrt{gk_{s}\tan\text{h}(k_{s}d)}-m'\sqrt{gk_{s}^{'}\tan\text{h}(k_{s}^{'}d)} \end{array}$$

Where *k̃_s_*,*k̃_s_* are the spatial wavevectors (with magnitudes *k_s_*, $k_{s}^{'}$) of the two shallow-water, first-order ocean waves interacting to produce the second-order backscatter. *m*, *m*′ are equal to +1, -1 for waves moving toward, away from the radar respectively.

The electromagnetic coupling coefficient has the same form as for deep water [5] but with shallow-water wavevectors:

(5) $$\Gamma_{\text{EM}}^{s}=0.5\;\left[\frac{({\tilde{k}}_{s}.{\tilde{k}}_{0})({\tilde{k}}_{s}^{\prime }.{\tilde{k}}_{0})/k_{0}^{2}-2{\tilde{k}}_{s}.{\tilde{k}}_{s}^{\prime }}{\sqrt{{\tilde{k}}_{s}.{\tilde{k}}_{s}^{\prime }}-k_{0}\Delta }\right]$$

where Δ is the normalized surface impedance. The hydrodynamic coupling coefficient, derived by Barrick and Lipa [6] through solution of the equations of motion and continuity, is a function of water depth:

(6) $$\Gamma_{H}^{s}=-\frac{i}{2}\;\left[k+k\prime -\frac{\left(kk\prime -{\tilde{k}}_{s}.{\tilde{k}}_{s}^{\prime }\right)}{mm\prime \sqrt{kk\prime }}\left(\frac{\omega^{2}+\omega_{B}^{2}}{\omega^{2}-\omega_{B}^{2}}\right)+\frac{\omega \left\{{\left(m\sqrt{gk_{s}}\right)}^{3}{\text{csch}}^{2}(k_{s}d)+{\left(m\prime \sqrt{gk_{s}^{\prime }}\right)}^{3}{\text{csch}}^{2}(k_{s}^{\prime }d)\right\}}{g(\omega^{2}-\omega_{B}^{2})}\right]$$

where k and k′ are the spatial wavenumbers of the scattering waves in deep water. The deep- and shallow-water spatial wavenumbers are related as follows:

(7) $$\begin{array}{cc} k=k_{s}\tan\text{h}(k_{s}d) & \;k\prime =k\prime_{s} \end{array}\tan\text{h}(k\prime_{s}d)$$

The total radar coupling coefficient Γ*^s^* is the coherent sum of the hydrodynamic and electromagnetic terms

(8) $$\Gamma^{s}=\Gamma_{EM}^{s}+\Gamma_{H}^{s}$$

It can be shown from these equations that at constant wavenumber, the coupling coefficient increases as the water depth decreases, resulting in an increasing ratio of second- to first-order energy as the depth decreases.

In the following analysis, we assume that the deep-water directional wave spectrum is spatially homogeneous and that any inhomogeneity in shallow water arises from wave refraction. When energy dissipation can be neglected, it follows from linear wave theory that since the total energy of the wavefield, is conserved, the shallow-water wave spectrum expressed in the appropriate variables is equal to the deep-water spectrum [7]:

(9) $$S_{s}({\tilde{k}}_{s})=S(\tilde{k}\;)$$

where the deep- and shallow-water wave vectors are related by Snell's law and the dispersion equation:

(10) $$k\cos(\theta +\beta )=k_{s}\cos(\theta_{s}+\beta )$$

(11) $$k=k_{s}\tan\text{h}(k_{s}d)$$

**Figure 1.**
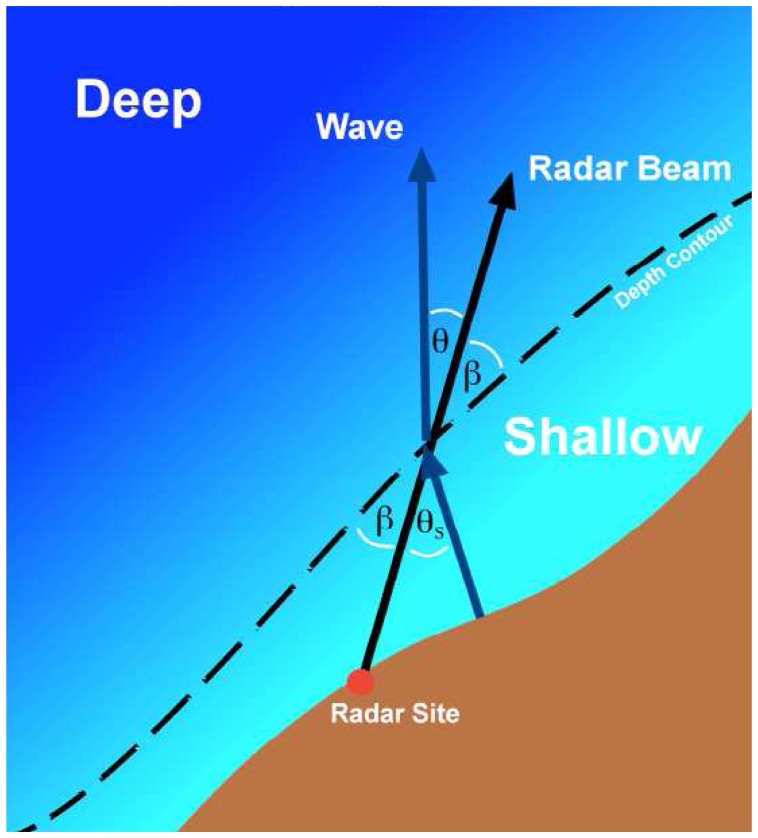
Schematic geometry of the radar beam and an ocean wave train at a depth contour, denoted by the dashed line. Wave angles are measured counter-clockwise from the radar beam to the direction the wave is moving. Increasing *θ_s_*, *θ* by 180° would define an incoming wave.

Here *β* is the angle between the radar beam and the depth contour and *θ_s_* ,*θ* are the angles between the radar beam and the shallow-, deep-water ocean waves respectively. Figure 1 illustrates refraction at a contour between regions of differing depth.

The shallow- and deep-water rms waveheights are given by:

(12) $$\begin{array}{cc} H^{2}=\int_{0}^{\infty }\int_{0}^{2\pi }S(k,\theta )\text{kdkd}\theta & H_{s}^{2}=\int_{0}^{\infty }\int_{0}^{2\pi }S_{s}(k_{s},\theta_{s})k_{s}dk_{s}d\theta_{s} \end{array}$$

Substituting (10) and (11) into (12) gives the following relations which are useful for deriving the shallow- from the deep-water wave spectrum and vice versa:

(13) $$\begin{array}{cc} H^{2}=\int_{0}^{\infty }\int_{0}^{2\pi }S_{s}(k_{s},\theta_{s})J(k_{s},\theta_{s}){\text{kdk}}_{s}d\theta_{s} & \;H_{s}^{2}=\int_{0}^{\infty }\int_{0}^{2\pi }S\left(k,\theta \right)J^{-1}(k_{s},\theta_{s})k_{s}\text{dk}\;d\theta \end{array}$$

where the Jacobian *J*(*k_s_*, *θ_s_*) is given by:

(14) $$J(k_{s},\theta_{s})=\left|\begin{array}{cc} {\left(\frac{\partial k}{\partial k_{s}}\right)}_{\theta } & {\left(\frac{\partial \theta }{\partial k_{s}}\right)}_{k}\\ {\left(\frac{\partial k}{\partial \theta_{s}}\right)}_{\theta } & {\left(\frac{\partial \theta }{\partial \theta_{s}}\right)}_{k} \end{array}\right|={\left(\frac{\partial k}{\partial k_{s}}\right)}_{\theta }{\left(\frac{\partial \theta }{\partial \theta_{s}}\right)}_{k}=\left[1+\frac{k_{s}d\sec\;h^{2}(k_{s}d)}{\tan\text{h}(k_{s}d)}\right]\;\left[\frac{\sin(\theta_{s}+\beta )}{\sin(\theta +\beta )}\right]$$

The first- and second-order radar cross sections in shallow water at frequency *ω* and azimuth angle *φ* are given by:

(15) $$\sigma_{s}^{1}\left(\omega ,\varphi \right)=k_{0}^{4}\sum_{m\prime =\pm 1}S_{s}(2k_{0},\varphi +(m\prime +1)\frac{\pi }{2})\delta (\omega -m\prime \omega_{B})$$

where *S_s_*(*k, α*)) is the directional ocean wave spectrum for wavenumber k and direction *α*□.

(16) $$\begin{array}{l} \sigma_{s}^{2}\left(\omega ,\varphi \right)=k_{0}^{4}\sum_{m,m\prime =\pm 1}\int_{0}^{2\pi }\int_{-\infty }^{\infty }|\Gamma_{s}^{2}|S_{s}(k_{s},\theta_{s}+\varphi +m\pi )\\ .S_{s}(k_{s}^{\prime },\theta_{s}+\varphi +m\prime \pi )\delta \left(\omega -m\sqrt{gk_{s}\tan\text{h}(k_{s}d)}-m\prime \sqrt{gk_{s}^{\prime }\tan\text{h}(k_{s}^{\prime }d)}\right)k_{s}dk_{s}\;d\theta_{s} \end{array}$$

where the coupling coefficient Γ*_s_* is given by (8). The values of m and m′ in (16) define the four possible combinations of direction of the two scattering waves. Common numerical multiplicative constants in (15) and (16) have been omitted. It can be shown from (4) that the wavenumbers of the scattering waves are related as follows:

(17) $$k\prime_{s}=\sqrt{k_{s}^{2}+2k_{s}\cos(\theta_{s})+1}$$

To compute the second-order integral in (16), we choose as integration variables *k_s_* and the deep-water angle *θ*. In terms of these variables (16) becomes

(18) $$\sigma_{s}^{2}\left(\omega ,\varphi \right)=k_{0}^{4}\sum_{m,m\prime =\pm 1}\int_{0}^{2\pi }\int_{-\infty }^{\infty }I(k_{s},\theta )\delta (\omega -h(k,\theta ))\left|{\left(\frac{\partial k_{s}}{\partial h}\right)}_{\theta }\right|\text{dhd}\theta$$

where

(19) $$h(k\;,\theta )=m\sqrt{\text{gk}}-m\prime \sqrt{gk_{s}^{\prime }\tan\text{h}(k_{s}^{\prime }d)}$$

and

(20) $$I(k_{s},\theta )=\left|\Gamma_{s}^{2}\right|S(k,\theta +\varphi +m\pi )S(k\prime ,\theta +\varphi +m\prime \pi )k_{s}{\left(\frac{\partial \theta_{s}}{\partial \theta_{s}\prime }\right)}_{k}$$

and where we have substituted (9) for the shallow water directional spectra. The factors ${\left(\frac{\partial \theta_{s}}{\partial \theta_{s}\prime }\right)}_{k}$ and ${\left(\frac{\partial k_{s}}{\partial h}\right)}_{\theta }$ are obtained by differentiation using (10), (11) and (19).

To calculate the integral in (18), it is first reduced to a single-dimensioned integral using the delta function constraint. The remaining integral is computed numerically.

Frequency contours are defined by:

(21) ω−h(k,θ)=0

which is solved for *k* as a function of *θ* for a given value of *ω*. Due to wave refraction, the shallow water angle and wavenumber have discontinuities when the deep-water wave moves parallel to the depth contour, i.e. when

(22) θ=−β,π−β

where *β* is the angle between the radar beam and the depth contour.

**Figure 2.**
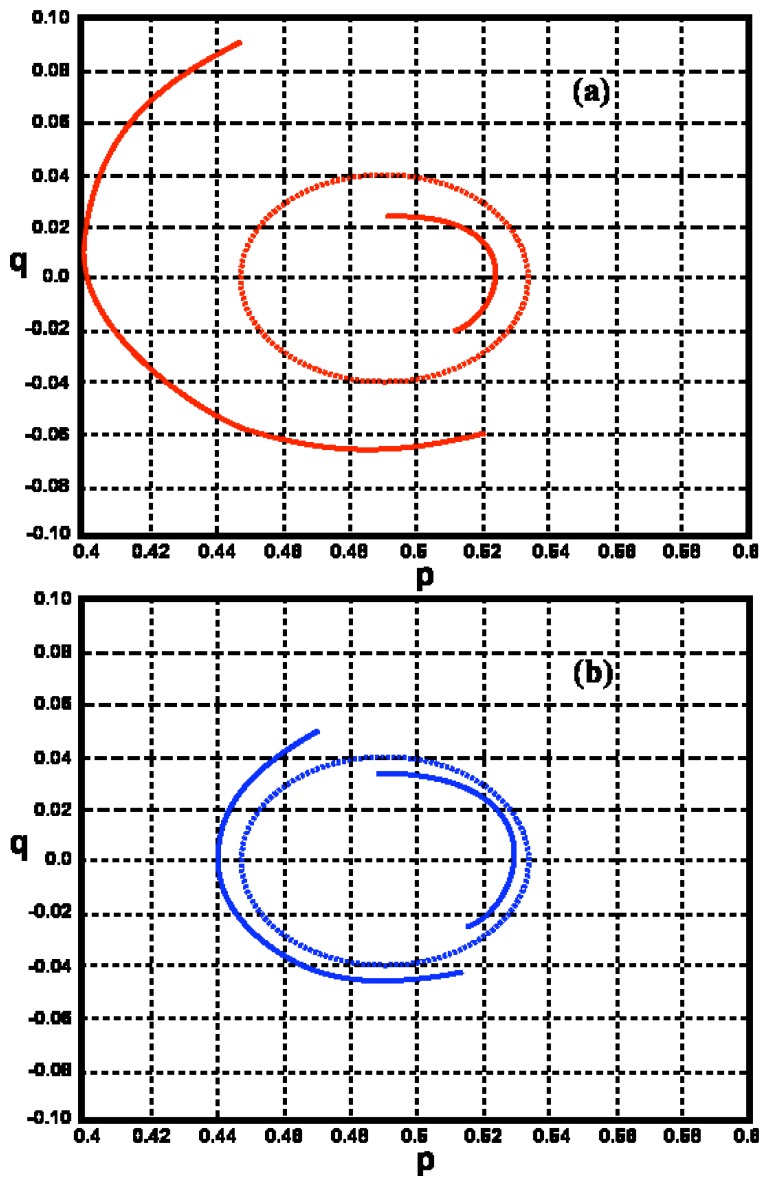
Examples of frequency contours for water of depth 10m (continuous lines) compared with the corresponding contours for deep water (dashed lines). Normalized frequency: *ω* / *ω_B_*=1.2, *β*=60deg. Radar frequency: (a) 5Mhz, (b) 25Mhz

Frequency contours are hence also discontinuous due to this effect at deep-water wave angles defined by (22). Examples of frequency contours for deep- and shallow-water are shown in Figure 2, plotted in normalized deep-water spatial wavevector space *k̃* / (2*k*_0_). Normalized components *p*, *q* are defined so that *p* is along the radar beam and *q* perpendicular:

(23) $$\begin{array}{lll} p & = & \left(k_{0}+k\cos(\theta )\right)/(2k_{0})\\ q & = & k\sin(\theta )/(2k_{0}) \end{array}$$

The discontinuities in the frequency contours are more pronounced when the contour is drawn in shallow-water wavenumber space, as it follows from (10), (11) that there are discontinuities in the shallow-water wave angle due to wave refraction.

It can be seen from Figure 2 that the deep-water ocean wave numbers corresponding to a given radar spectral frequency change with depth: they become either greater or smaller than the deep-water values, depending on the wave direction. This results in the frequency of second-order peaks in the radar spectrum changing with water depth.

**Figure 3.**
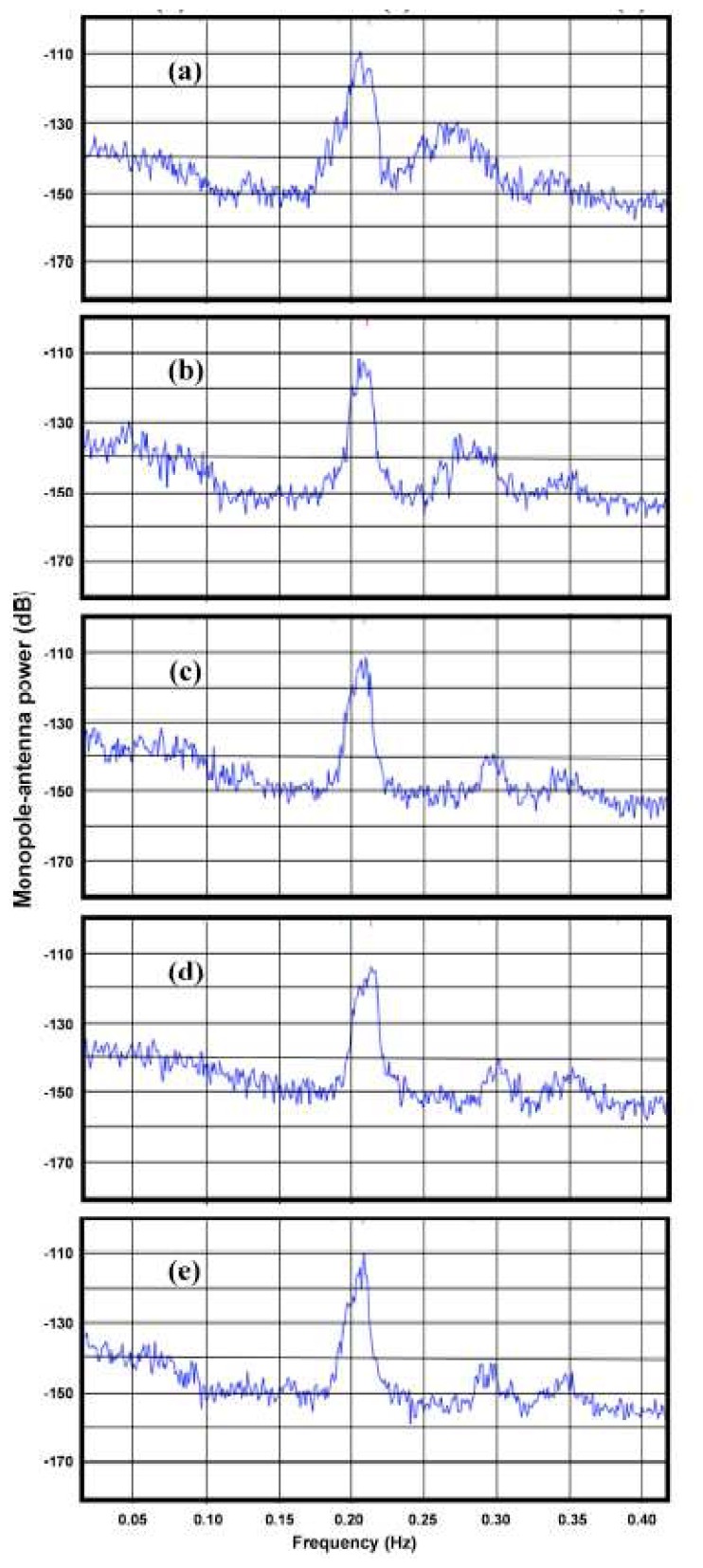
Spectra from a 5MHz SeaSonde monopole antenna. Range/ Water depth: (a) 18km/ 5 -20m (b) 30km/10-50m (c) 42km/ 20-70m (d) 48km/ 35-80m (e) 54km/ 40-100m

The effects of shallow-water on measured radar spectra are illustrated in Figure 3, which shows measured spectra from a 5 MHz radar in five radar range cells, with distances ranging from 18km to 60km. As the water depth decreases, the second-order energy increases relative to the first-order and the frequency displacement between the first- and second-order peaks decreases. In the outer ranges, the second-order structure is almost the same from range cell to range cell, as the water is effectively infinitely deep.

## 3. Narrow-beam radar spectral simulations

To gain insight into the effects of shallow water, simulated radar echo spectra were calculated for a narrow-beam radar, using the model directional wave spectrum defined in [8] which consists of the sum of two terms: a continuous high-frequency wind wave spectrum and a swell component that is an impulse function in both wavenumber and direction. The swell component is defined by

(24) $$S_{s}(k_{s},\theta_{s})=H_{s}^{*2}\delta (\theta_{s}-\theta_{s}^{*})\delta (k_{s}-k_{s}^{*})$$

Where $H_{s}^{*}$ , $\theta_{s}^{*}$, $k_{s}^{*}$ are the specified rms waveheight, direction and wavenumber. For this model, four sharp spikes occur in the radar spectrum. Here we consider only the second-order sideband for which m=1, m′=1, and assume water depths in the range 5-100m and radar transmit frequencies of 5Mhz and 25Mhz. For these values, it can be shown numerically that Doppler frequencies are always greater than the positive Bragg frequency. The radar beam is taken to be pointing perpendicular to parallel depth contours (i.e *β* = 90° in Figure 1)

### 3.1 Effect of water depth on waveheight

For our model it follows from (13) that the relationship between the shallow- and deep-water rms waveheights is given by:

(25) $$H_{s}^{*}=H^{*}\sqrt{\frac{\sin(\theta^{*}+\beta )/\sin(\theta_{s}^{*}+\beta )}{\left[\tan\text{h}(k_{s}^{*}d)+k_{s}^{*}d\sec h^{2}(k_{s}^{*}d)\right]}}$$

**Figure 4.**
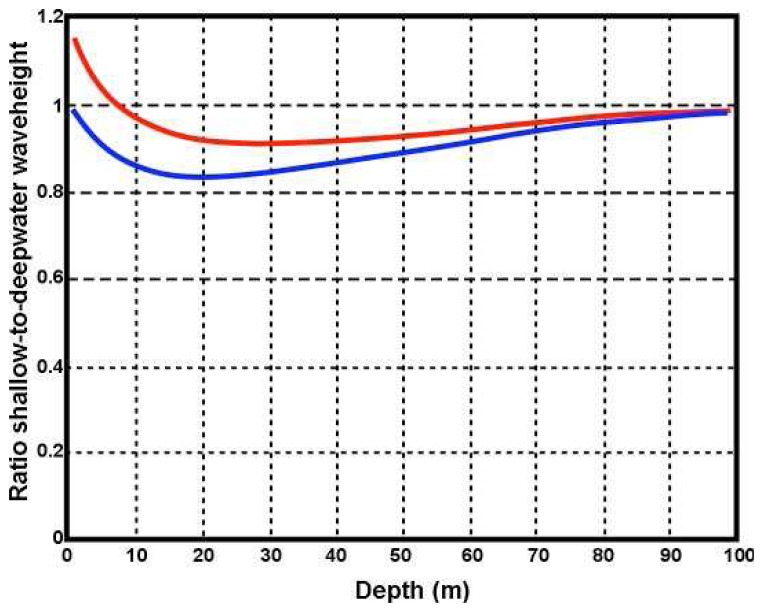
The ratio of shallow- to deepwater waveheight plotted vs. depth for a 12 s wave. Wave direction in deep water relative to the radar beam: Red 180°, Blue 135°

This relationship is of course independent of radar frequency and has many angle symmetries. Figure 4 shows the ratio plotted as a function of depth for different wave directions.

It can be seen from Figure 4 that the waveheight initially decreases with decreasing depth as the wave enters shallow water but increases at depths below about 20m, which agrees with [7].

### 3.2 Effect of water depth on Doppler shifts

**Figure 5.**
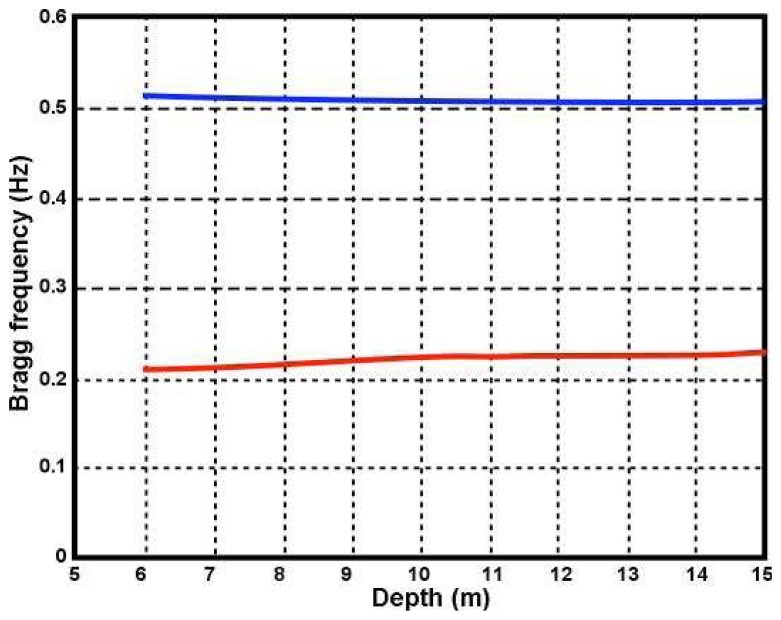
Bragg frequency plotted as a function of depth. Radar transmit frequency: Red 5Mhz, Blue 25Mhz

It follows from (3) that for a given radar frequency, the Bragg frequency decreases with depth, causing the Bragg peaks to move slightly closer together. Figure 5 shows the Bragg frequency plotted as a function of depth.

It can be seen from Figure 5 that the change in the Bragg frequency with depth is small.

**Figure 6.**
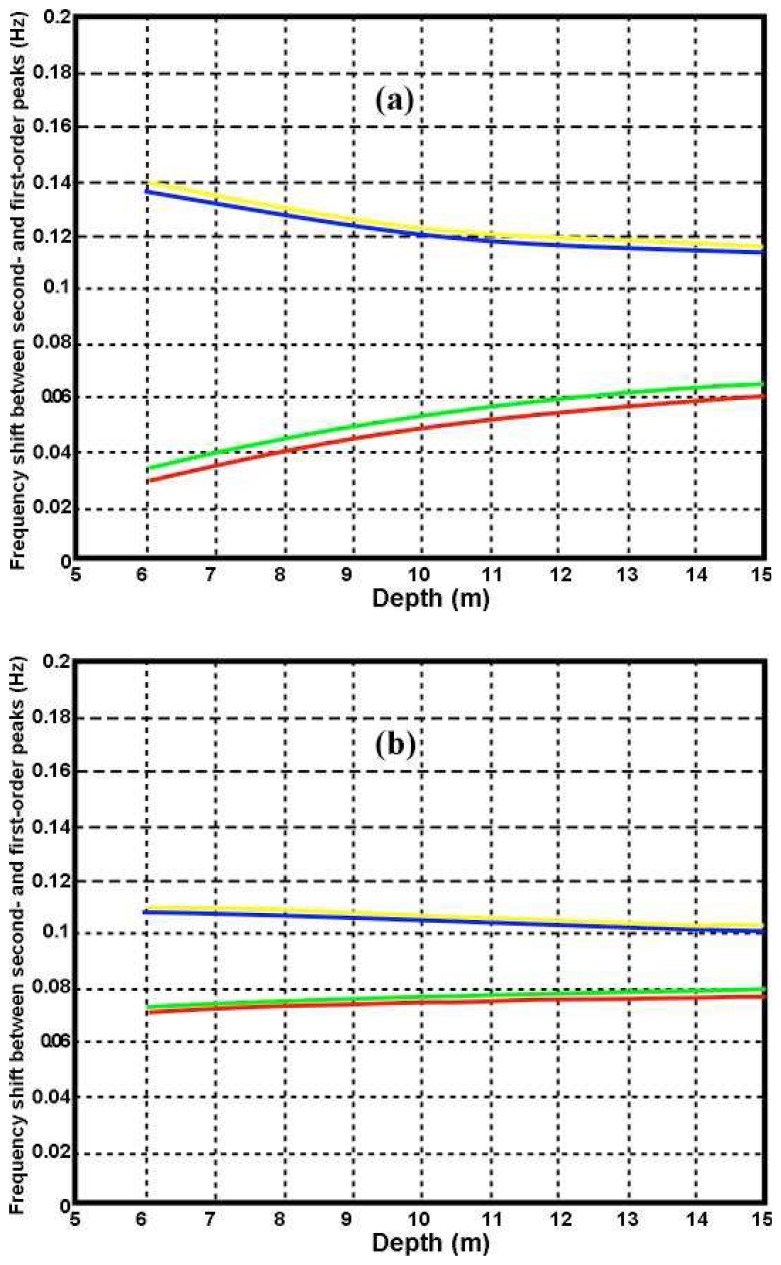
The frequency shift of the second-order peak from the Bragg frequency for an 11s wave. (a) 5Mhz (b) 25Mhz. Angle between wave and radar beam: Yellow 0°, Blue 45°, Green 135°, Red 180°

Figure 6 shows the displacement of the second-order peak from the Bragg frequency plotted as a function of depth for an 11s wave moving at different angles with respect to the radar beam.

It can be seen from Figure 6 that as the water depth decreases, the second-order peak shifts toward the Bragg frequency for waves moving toward the radar, and further away for waves moving away from the radar. This is consistent with the two branches of the contour plot as shown in Figure 2. This effect is more marked for lower radar frequencies and can be seen in the measured spectra shown in Figure 3 in which the second-order peak moves closer to the first-order as the range from the radar and water depth decrease, with waves moving toward the radar.

### 3.3 Effect of water depth on radar spectral amplitudes

**Figure 7.**
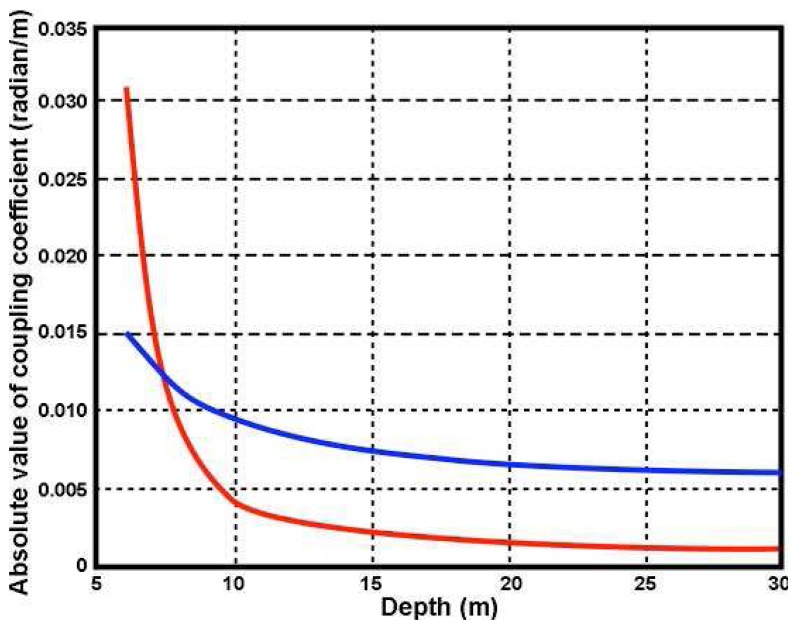
The absolute value of the coupling coefficient Γ*_s_* vs. depth for a 9 sec wave. Radar frequency: Red: 5Mhz, Blue: 25Mhz.

**Figure 8.**
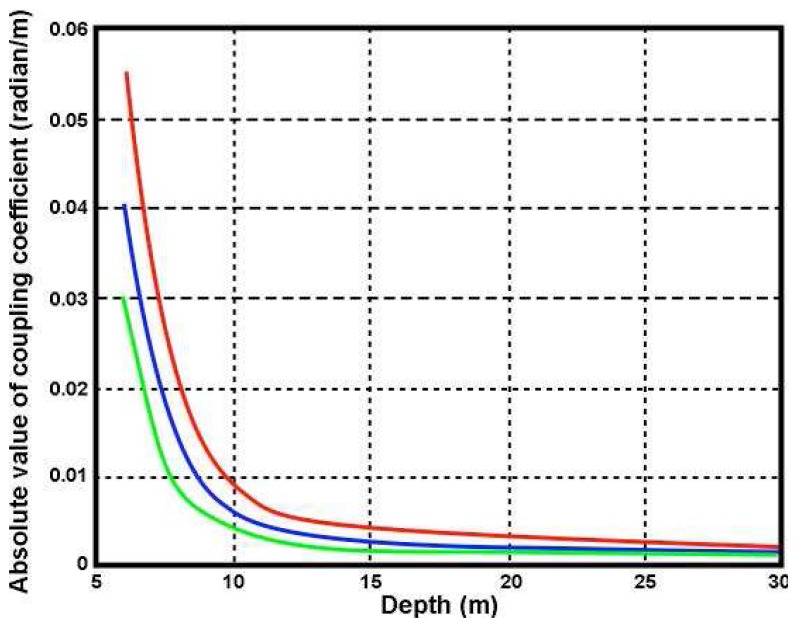
The absolute value of the coupling coefficient vs. depth for waves of different period. Radar transmit frequency: 5Mhz. Wave period: Red 15s, Blue 12s. Green 9s.

It is shown in [8] that for the impulse-function model defined by (24), the ratio *R* of the second-order to first-order energy is given by:

(26) $$R=2H_{s}^{*2}\left|\Gamma_{s}^{2}\right|$$

where the coupling coefficient Γ*_s_* is evaluated at wavevectors defined by $\theta_{s}^{*}$, $k_{s}^{*}$. Γ*_s_* increases with decreasing depth and increasing wave period at a given radar frequency as illustrated in Figures 7 and 8, which also show that shallow water has a greater effect as the radar transmit frequency decreases.

**Figure 9.**
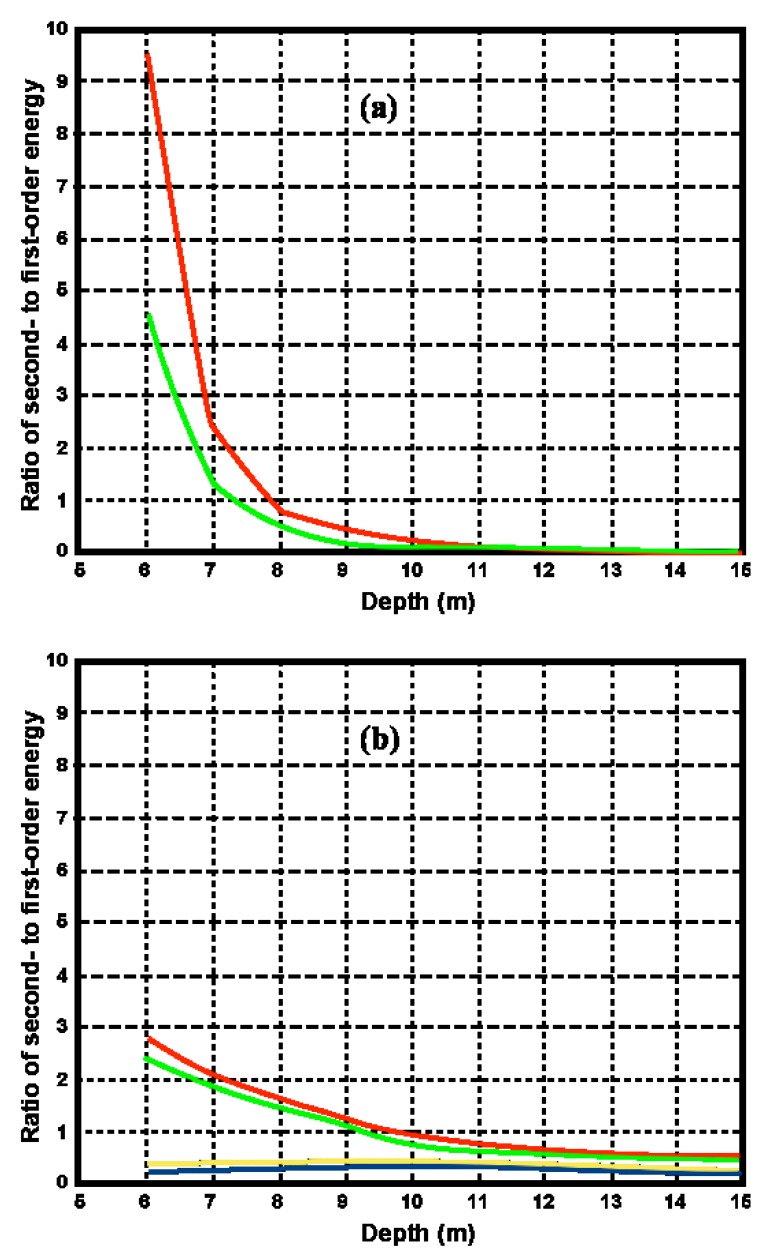
Ratio of second - to first-order energy for an 11s wave. Significant waveheight: 2.4m. Radar transmit frequency: (a) 5 Mhz, (b) 25 Mhz. Angle between wave and radar beam: Yellow 0°, Blue 45°, Green 135°, Red 180°

Since the coupling coefficient increases as the depth decreases, it follows from (26) that the second-order energy will increase with respect to the to first-order. This effect can be seen in the measured radar spectra shown in Figure 3. Figure 9 shows the theoretical ratio of the second- to the first-order energy obtained from (26) using our model for an 11s wave.

It can be seen from Figure 9 that the ratio of the second- to the first-order energy exceeds unity (i.e. the calculated second-order energy exceeds the first-order energy) for depths less than about 8 m for a 5MHz transmit frequency and for depths less than about 10m for a 25 MHz transmit frequency.

This subsection demonstrates an important point. Since we have shown that the waveheight itself actually decreases slightly upon moving into shallow water, while the second-order echo increases significantly due to the rapid growth of the coupling coefficient, wrongly using deep-water inversion theory to estimate waveheight will overestimate this important quantity. We note that all previous treatments and demonstrations of wave extraction have been based on deep-water theory, even when in fact many of the radar observations have been made in shallow water.

### 3.4 Effect of water depth on breakdown of theoretical model

When the magnitude of the second-order energy approaches that of the first-order, it is apparent that the perturbation expansions on which (15) and (16) are based are failing to converge and they therefore cannot provide an adequate description of the radar echo. This effect is similar to the well known radar spectral saturation occurring when the waveheight exceeds a limit defined by the radar transmit frequency. Above this waveheight limit, the radar spectrum loses its definitive shape and the perturbation expansions fail to converge. The deep-water saturation limit on the significant waveheight *W_Sat_* (defined to be four times the rms waveheight) is given approximately by the relation:

(27) $$W_{\text{Sat}}=2/k$$

For shallow-water, the saturation of the radar spectrum is exacerbated by the increase of the coupling coefficient and the radar spectrum saturates for waveheights less than that defined by (27). We here define the shallow-water saturation limit $W_{\text{Sat}}^{s}$ for the model to be that waveheight for which the second-order energy equals the first-order, and the ratio R is given by:

(28) R=1

**Figure 10.**
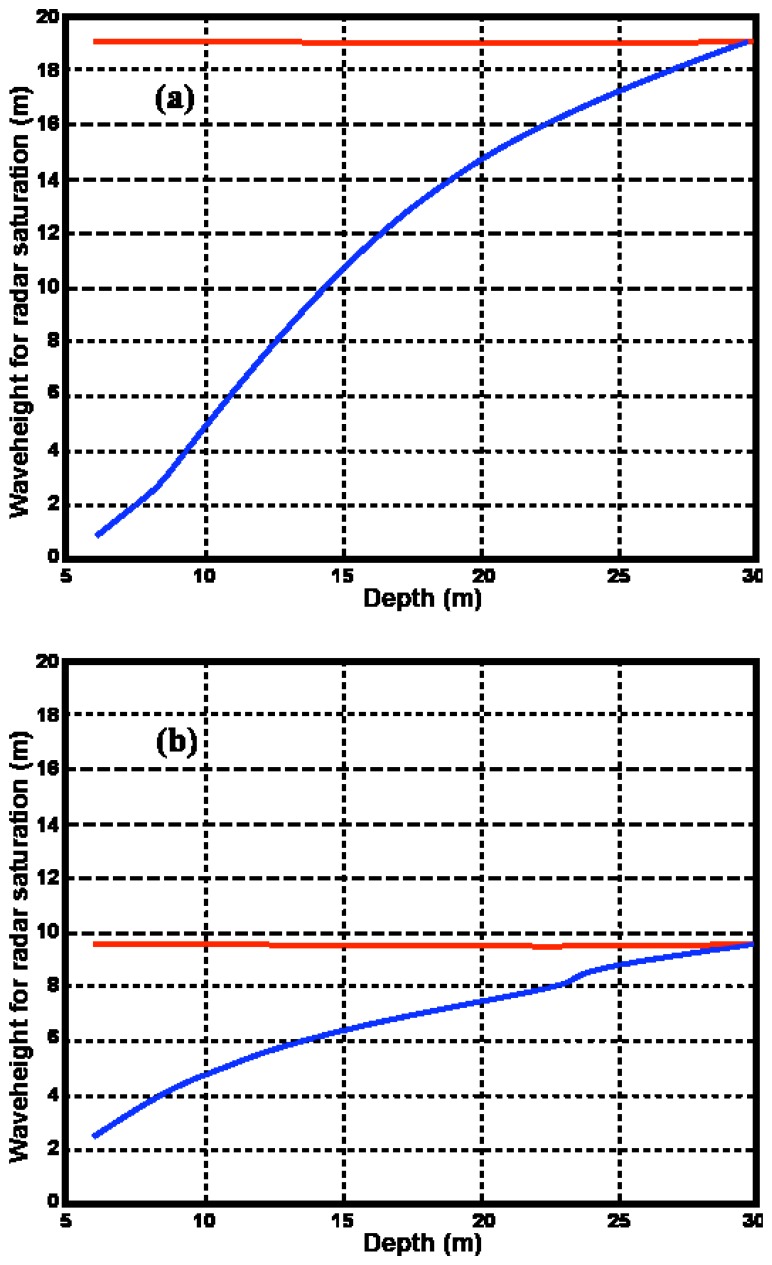
Significant waveheight saturation limits for an 11-second wave coming straight down the radar beam. Radar transmit frequency: (a) 5 Mhz , (b) 25 Mhz Red: deep-water saturation limit *W_Sat_*, Blue: shallow-water saturation limit $W_{\text{Sat}}^{s}$

In practice the theory may fail before this limit is reached. *W_Sat_* and $W_{\text{Sat}}^{s}$ are plotted vs. depth in Figure 10 for two different radar frequencies. At depths of 30m the saturation limits are approximately equal. At depths less that 30m, the shallow-water limit drops off sharply, particularly for the lower transmit frequency. Thus the radar spectrum can be expected to saturate at lower values of waveheight in shallow water.

For waveheights above the saturation limit, the waveheight predicted by the theory will be too high. However the theory cannot be applied at all when the second-order spectrum merges with the first, as then separation is not possible.

### 3.5 Depth limits for significant shallow-water effects

**Figure 11.**
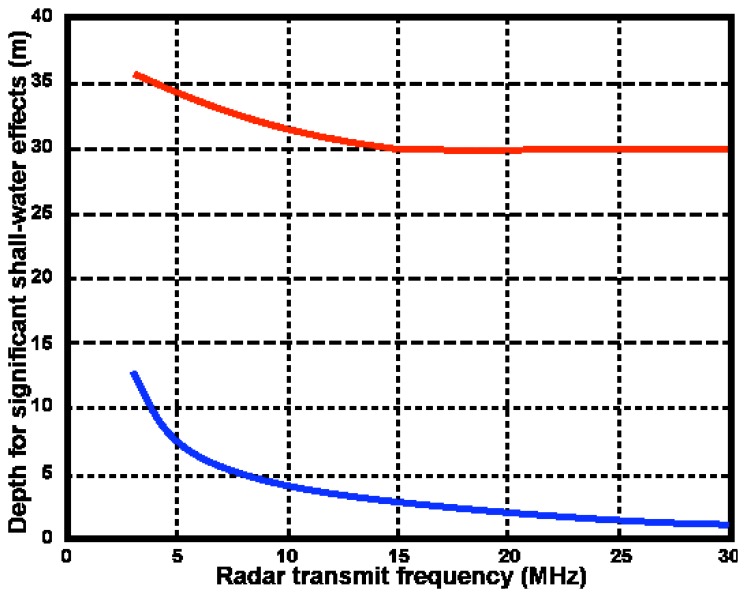
Depths at which shallow-water effects become significant vs. radar transmit frequency. Red: second-order echo. Blue: First-order echo.

We estimate depths for which shallow-water effects become significant as follows: For first-order echo, the depth limit is defined by equality in (1). At this depth, the Bragg frequency defined by (3) is 96% of its deep-water value. For second-order echo, we define the depth limit *D^S^* at which shallow-water effects become significant as the value at which the coupling coefficient defined by (8) exceeds 1.25 times the deep-water value. Figure 11 plots the depths *D^S^* vs radar transmit frequency for an 11s wave.

Figures 10 and 11 help in assessing the validity of the existing deep-water methods. However they are based on a wave model (24), which is quite restrictive: waves of a single wavelength are assumed to come down the radar beam. Also Figure 11 applies only to an 11s wave. Performing similar studies for more general wave spectral models is beyond the scope of this paper. However, we observe that: (a) Shallow water effects are stronger for longer ocean wavelengths (b) Second-order radar spectra for m=1, m′=1are strongest for waves down the radar beam. (c) The stronger the second-order energy for a given waveheight, the sooner the radar spectrum will saturate as waveheight increases. Therefore shallow-water effects will be more marked at a given waveheight for a broad nondirectional spectrum that includes longer wavelengths e.g. the Pierson Moskowitz model (32). These differences would probably not be large however, due to the sharp cutoff of wave-spectral models for long wavelengths. The opposite effects would be expected for spectra that include wave directions not directly down the radar beam e.g. a cardioid directional distribution. To summarize these effects: $W_{\text{Sat}}^{s}$ will be less and *D^S^* will be greater than the values shown in Figs. 10 and 11 for the following changes from the wave spectrum (24): broad nondirectional spectrum, wave period > 11s. $W_{\text{Sat}}^{s}$ will be greater and *D^S^* will be less for broad directional distributions, waves nonparallel to radar beam , wave period < 11s.

## 4. Application to measured data

### 4.1 Data set

**Figure 12.**
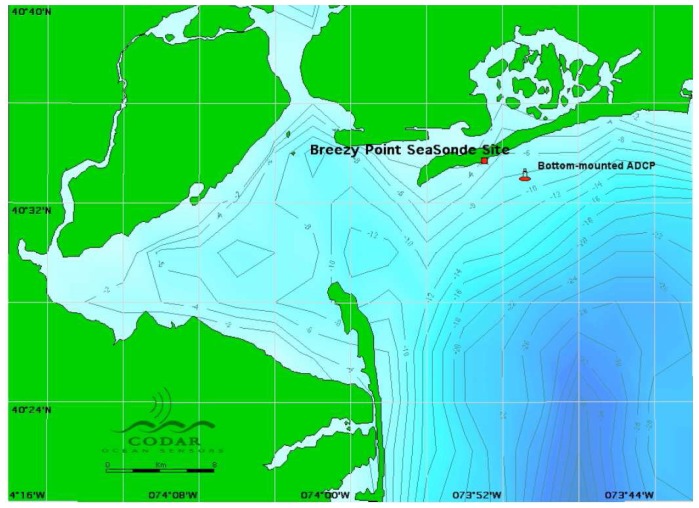
The coastline and bathymetry (contours in meters) around Breezy Point, New Jersey, showing the positions of the SeaSonde and the bottom-mounted ADCP.

The results presented here are based on analysis of 10-minute radar spectra measured by a 25MHz SeaSonde located at Breezy Point, NJ. The time period from December 29 to 30, 2005, was chosen because simultaneous coverage provided by the SeaSonde and a bottom-mounted ADCP allowed a direct comparison to be made between results from the two sensors. The ADCP was located in the second radar range cell in water of depth 8m. The bathymetry in the area and the locations of the two sensors are shown in Figure 12.

In our analysis, depth contours near the radar are assumed to be parallel to shore and the depth profile is obtained from Figure 12.

**Figure 13.**
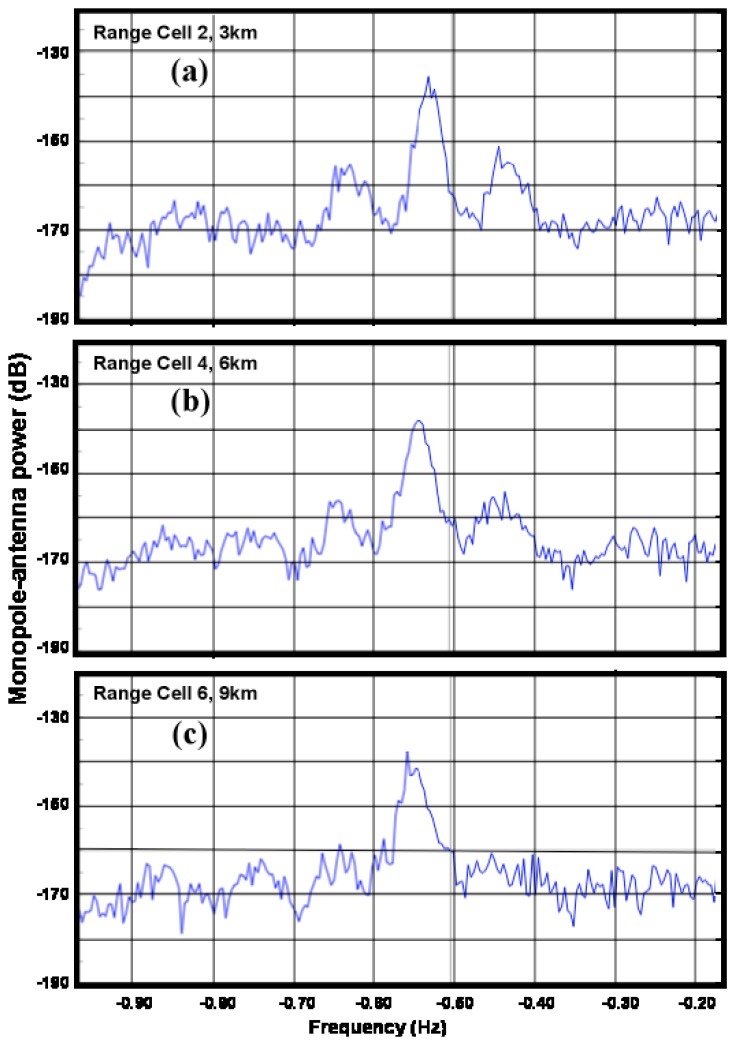
(a) Spectra measured by the 25MHz SeaSonde at Breezy Point. at 1:00pm 12/30/2005. Range: (a) 3 km (b) 6 km (c) 9 km.

Figure 13 shows measured spectra from the Breezy Point SeaSonde at three ranges: the second-order energy can be seen to increase relative to the first-order as the water depth decreases.

### 4.2 Interpretation of the radar spectra

Lipa and Barrick [5] describe the extension of the narrow-beam theory described in Section 2 to apply to a broad antenna system such as the SeaSonde, assuming ideal antenna patterns. From the antenna voltage cross spectra, we form as intermediate data products the first five Fourier angular coefficients of the broad-beam return over a selected range ring surrounding the radar. These coefficients, designated by the index n = -2, -1, 0, 1, 2, are defined in terms of the narrow-beam first and second-order return through the relation:

(29) $$b_{n}^{1,2}(\omega )=\int \sigma_{s}^{1,2}(\omega ,\varphi )tf_{n}(\varphi )d\varphi$$

where the integration over azimuth angle *φ* is performed over open water around the radar range cell and the superscripts refer to first- and second-order respectively. The narrow-beam radar cross sections $\sigma_{s}^{1,2}\;(\omega ,\varphi )$ are defined in terms of the ocean wave spectrum by (15), (16). Following the notation in [5], the trigonometric functions *tf_n_*(*φ*) are given by

(30) $$\begin{array}{lll} tf_{n}(\varphi ) & = & \sin(n\varphi )\;n<0\\ & = & \cos(n\varphi )\;n\geq 0 \end{array}$$

As described in [4], there are three steps in the interpretation of the radar spectrum to give deep-water wave information.

a) The first- and second-order regions are separated.
b) The first order region is analyzed to give the ocean wave spectrum at the Bragg wavenumber. It is assumed that deep-water theory is adequate for this step, as Bragg waves are short and hence insensitive to the effects f shallow water, see Figure 5.
c) Second-order radar spectral data is collected from the four second-order sidebands of 10-minute averaged cross spectra and fit to a model of the deep-water ocean wave spectrum. Least-squares fitting to the radar Fourier coefficients is used to derive estimates of the significant wave height, centroid period and direction. During this step, the second-order spectrum is effectively normalized by the first-order, eliminating unknown multiplicative factors produced by antenna gains, path losses etc.
  Shallow-water analysis requires a further step:
d) The shallow-water wave spectrum is calculated from the deep-water spectrum using (9)–(11).

### 4.3 Model ocean wave spectrum

For our analysis, we define a model for the deep-water ocean wave spectrum as the product of directional and nondirectional factors:

(31) $$S(k,\varphi )=Z(k)\cos^{4}\left(\frac{\varphi -\varphi *}{2}\right)$$

The directional factor in (31) has a cardioid distribution around the dominant direction φ*. For describing the second-order spectrum, φ* is taken to be the dominant long-wave direction. For describing the first-order spectrum, φ* is the short-wave direction, which is assumed to be the same as the wind direction. For the nondirectional spectrum we use the Pierson-Moskowitz model Z(k):

(32) $$Z(k)=\frac{Ae^{-0.74{\left(k_{c}/k\right)}^{2}}}{k^{4}}$$

whose parameters are the cutoff wavenumber *k_c_* and a multiplicative constant A. The waveheight, centroid period and direction can be defined in terms of the model parameters. The significant waveheight follows from the directional spectrum through the relation:

(33) $$W=4{\left(\int_{0}^{\infty }\int_{\gamma_{1}}^{\gamma_{2}}S(k,\varphi )\text{dkd}\varphi \right)}^{1/2}$$

This model has proven satisfactory for use in deep-water wave extraction software that produces waveheight, period, and direction. It has been used for real-time SeaSonde systems for many years, providing good agreement with in-situ measurements e.g. as shown in [4].

### 4.4 Results

**Figure 14.**
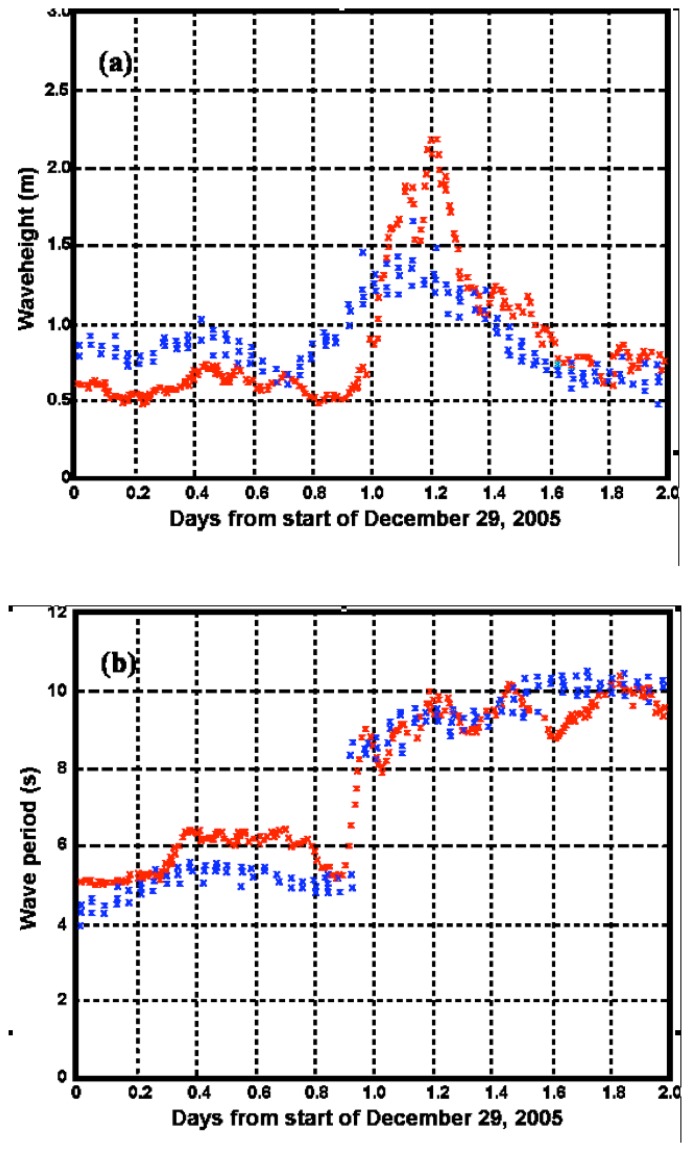

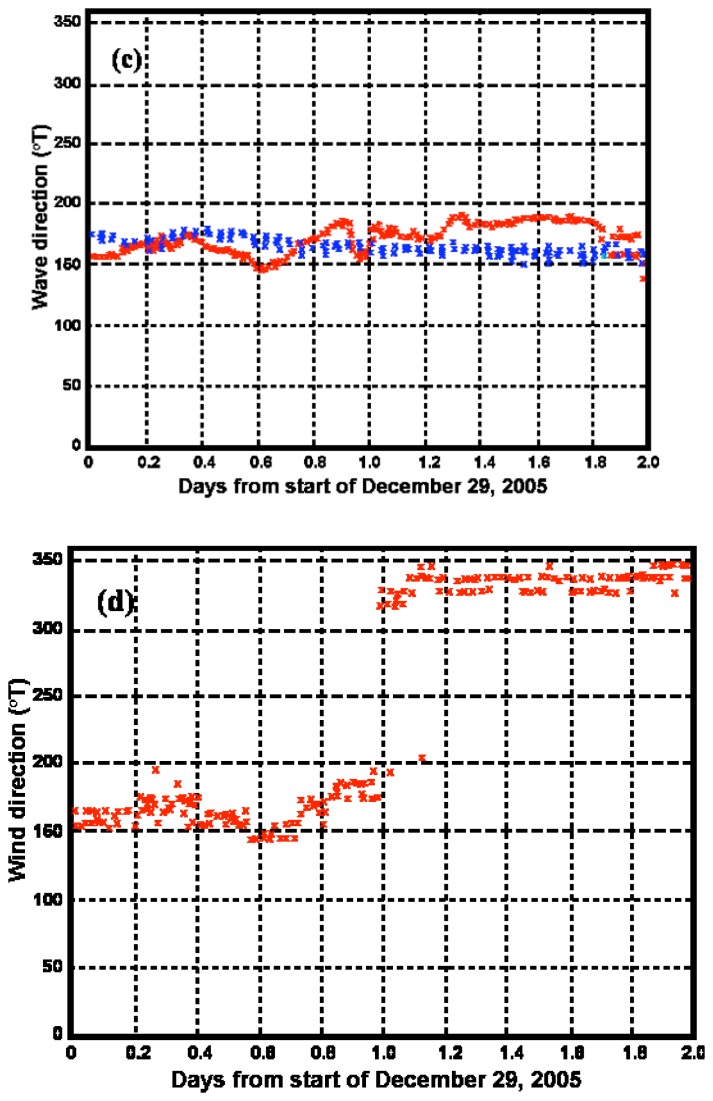
SeaSonde (red) and ADCP (blue) results for (a) Significant waveheight (b) Wave period (c) Wave direction (d) Wind direction.

Figure 14 shows SeaSonde results in the second radar range cell calculated using the shallow-water theory described above, together with the ADCP results.

It can be seen from Figure 14 that southerly winds veer to the northwest after the passage of a storm front. Subsequently the wave height and period increase suddenly. Spectral saturation may be occurring at the peak of the storm, causing overestimates in the waveheight. Wave direction remains about the same, as due to wave refraction, wave directions in very shallow water are nearly perpendicular to the depth contours. Both radar and ADCP are observing directions in shallow water, hence this perpendicular condition is being enforced on the longer waves, although the wind direction driving short waves is seen to change significantly over this storm period.

Table 1 gives the bias and standard deviation between the SeaSonde and ADCP measurements of waveheight, wave period and direction, for the short-period waves before the storm and the longer-period waves afterwards.

**Figure 15.**
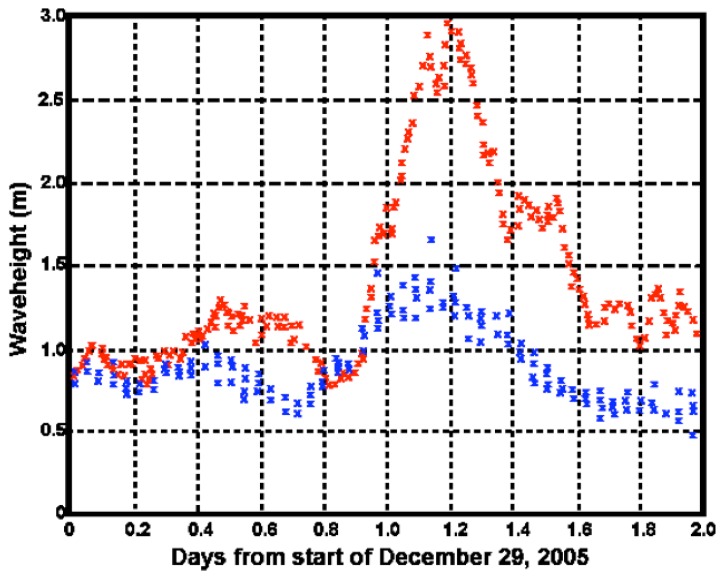
Significant waveheight: Red: SeaSonde calculated assuming infinite water depth. Blue: ADCP.

To emphasize the necessity of taking shallow water into account for this location, we estimated the waveheight assuming infinitely deep water. Figure 15 shows the SeaSonde results together with the ADCP waveheight. Clearly waveheight is overestimated with this assumption. The simulations described in Section 3 indicate that the cause of this overestimate is the failure to account for the increase of the coupling coefficient in shallow water.

Table 2 gives the bias and standard deviation between the SeaSonde and ADCP waveheight measurements, with the former calculated assuming infinitely deep water.

## 5. Conclusion

We have presented the theory of narrow-beam HF radar sea-echo from shallow water and illustrated the effect of decreasing water depth using simulations for a simple swell model of the ocean wave spectrum. The second-order spectral energy increases relative to the first-order as the water depth decreases, resulting in spectral saturation when the waveheight exceeds a limit defined by the radar transmit frequency. This effect is particularly marked for lower radar transmit frequencies. For waveheights above the saturation limit, the perturbation expansions on which Barrick's equations (15), (16) are based fail to converge. The saturation limit on waveheight is less for shallow water. Shallow water affects second-order spectra (which gives wave information) far more than first-order (which gives information on current velocities). Figure 11 shows the depths at which shallow-water effects become significant plotted as a function of radar frequency for an 11s wave. We discuss how the waveheight and depth limits would change for a more general model.

The shallow-water theory was then extended to apply to broad-beam systems such as the SeaSonde and applied to the interpretation of two days of radar data measured by a 25Mhz SeaSonde located on the New Jersey shore. During the measurement period, a storm passed over the area. An ADCP was operated in the second radar range cell in water 8m deep. Radar results were compared with simultaneous ADCP measurements. The comparison confirms aspects of the theory presented in Section 3. For the longer period waves occurring after the passage of the storm front, the standard deviation between SeaSonde and ADCP waveheight measurements decreased by a factor of three when the effects of shallow water were included in the analysis, and the bias decreased by a factor of five. Possible explanations for the remaining discrepancies are (a) the assumption of parallel depth contours (b) the assumption that the wave spectrum is homogeneous in the circular radar range cell (c) saturation in the radar spectrum around the peak of the storm, which, as discussed in Section 3, leads to the over-prediction of the waveheight.

## Figures and Tables

**Table 1.** Comparison statistics, radar vs. ADCP.

	Before storm	After storm
**Waveheight**		
Standard deviation	0.25m	0.35m
Bias	-0.23m	0.17m
**Wave Period**		
Standard deviation	0.76s	0.60s
Bias	0.70s	-0.27s
**Wave Direction**		
Standard deviation	13.8°	19.7°
Bias	-9.5°	17.0°

**Table 2.** Comparison statistics, radar vs. ADCP assuming deep water.

	Before storm	After storm
**Waveheight**		
Standard deviation	0.25m	0.95m
Bias	0.19m	0.90m
